# The acoustic change complex as a diagnostic tool for cochlear dead regions evaluated in normally hearing adults

**DOI:** 10.1038/s41598-025-02093-w

**Published:** 2025-07-07

**Authors:** Anna Schelenz, Emanuele Perugia, Lin Wu, Ewa Skrodzka, Karolina Kluk

**Affiliations:** 1 Department of Acoustics, Faculty of Physics, Adam Mickiewicz University, 61-614 Poznań, Poland; 2 Manchester Centre for Audiology and Deafness, University of Manchester, Manchester, M13 9PL UK

**Keywords:** Acoustic change complex, Dead regions, Objective test, Threshold-equalizing noise (TEN) test, Auditory system, Diagnosis

## Abstract

The loss of inner hair cells and/or neurons in the cochlea leads to cochlear dead regions (DRs). One of the consequences of DRs is noisy transmission of information from the cochlea to the brain, which results in poorer than expected perception of speech in noise. DRs can be detected using behavioural masking techniques such as the threshold-equalizing noise (TEN) test. However, the TEN test, although fast and easy to perform, requires participants’ active cooperation and thus is not suitable for use with infants, young children and adults who cannot provide behavioural responses. Recently, Kang et al. (2018) proposed the electrophysiological Acoustic Change Complex (ACC) as an objective test for diagnosing cochlear DRs, but with limited evidence supporting their proposed approach. Thus, the first step towards addressing this gap is to develop and assess an objective ACC detection method. Here, we evaluated three methods of detecting the ACC based on: i) the root mean square of N1-P2 amplitude as reported in Kang et al. (2018); ii) Signal-to-Noise Ratio (SNR) as recommended by the British Society of Audiology; and iii) bootstrap by multiple resampling of the original data in a random manner and calculating a measure of variance in the N1-P2 amplitude to noise floor ratio from the resampled data. We have also examined the relationship between ACC amplitude, threshold, and stimulus frequency/intensity. Twenty-three normally hearing adults were tested. The ACC was evoked by either a 1-kHz or 4-kHz pure tone presented simultaneously with the TEN at SNRs between 0 and 15 dB, in 3 dB steps relative to the TEN level. We found that the bootstrap method was most efficient at detecting ACC and was two times faster to administer than the commonly used British Society of Audiology method. We also found that the threshold of ACC detection is frequency dependent, i.e., the higher the frequency, the higher the signal level needed to evoke ACC. Thus, frequency-dependent ACC norms need to be established before this method can be used for detection of cochlear DRs in children and adults who cannot provide behavioural responses.

## Introduction

The use of Auditory Late Response (ALR) or Cortical Auditory Evoked Potential (CAEP) to estimate hearing thresholds in adults and older children is well established in clinical applications^1,2^. The ALRs are elicited by the onset of transient stimuli, such as pure tones, tone bursts or speech tokens^2–6^. A type of ALR is the Acoustic Change Complex (ACC), which is elicited by a change within an ongoing acoustic stimulus after the onset^7,8^. The ACC can be evoked by any changes in acoustic stimuli, such as in frequency^9,10^, intensity^10,11^, spectrum and amplitude^8^, interaural phase inversion^12^, or signal-to-noise ratio (SNR) of a background noise^13^. Irrespective of the stimulus, the ALR/ACC waveform is characterised by a series of peaks and troughs that are labelled P1, N1 and P2, which occur at about 50, 100 and 200 ms after the stimulus onset/change^1,2^. ACCs have been successfully recorded from normally hearing and hearing-impaired adults and children (e.g., ^14,15^).

Recently, Kang and colleagues^16^ proposed that ACC could be used as an objective test for diagnosing cochlear dead regions (DRs), i.e. regions in the cochlea where inner hair cells (IHCs) and/or neurons function so poorly that the tone falling within that region can only be detected via off-frequency listening^17,18^.

DRs may have an effect on speech perception and hearing-aid fitting. Participants with DRs performed worse on speech-in-noise tasks than participants without DRs^19,20^. The benefit of hearing-aid amplification for participants with DRs at high frequencies was questioned. It has been shown that the performance of a speech intelligibility task, measured as a function of low pass filtering of speech tasks, improved as a function of the filter cut-off up to about 1.5–2 times the edge frequency of the DR and then remained constant, as higher frequency information was not used^21,22^. The benefit of hearing-aid amplification at high frequencies is even more limited during speech-in-noise tasks^23,24^.

The off-frequency listening phenomenon provides the basis for cochlear DR diagnostic tests^17,25^. Hitherto, it has been possible to diagnose cochlear DRs only using behavioural techniques such as threshold-equalising noise (TEN) test^18,26^, and fast psychophysical tuning curves^27^, which require patient cooperation and, as such, are not appropriate for testing infants, young children and adults who cannot provide behavioural responses. To address this clinical need, several researchers have proposed the use of electrophysiological techniques to detect cochlear DRs^16,28–30^. Markessis et al.^28^ showed that it is possible to measure frequency tuning curves (FTCs) using auditory steady-state responses (ASSRs) in normally hearing adults. However, their proposed method required high masker levels and thus would be too high to be safe in people with significant hearing loss. An alternative approach was proposed by Wilding et al.^30^ to record ASSR response amplitude curves (RACs) using fixed-level signal and maskers, thus eliminating the requirement of high masker levels. They showed that it was possible to record repeatable RACs in normally hearing adults taking 32 min to record one RAC, but the method showed some inter-subject variability and needs to be tested in hearing-impaired participants with DRs. Another approach was proposed by Kang et al.^16^, who recorded ACCs in the background of TEN in both normally hearing and hearing-impaired adults.

The TEN is designed to produce almost equal masked thresholds in dB HL over the range of frequencies from 0.5 to 4 kHz for normally hearing participants and for participants with hearing impairment but without cochlear DRs^26,31^. In participants with a DR two criteria need to be met^26,31^: 1) the masked threshold needs to be 10 dB or more above the TEN(HL) level and 2) the masked threshold needs to be 10 dB or more above the absolute threshold. Since the TEN test is a clinical test, it is based on the single participant thresholds measured at several frequencies.

Kang et al.^16^, adopted the behavioural TEN(HL) test paradigm^26^ to develop an electrophysiological equivalent. In their paradigm, the TEN(HL) was presented at a fixed level with a pure tone presented in the ongoing TEN(HL) at several levels, i.e., SNR was varied. The ACC was elicited by the pure tone in the ongoing TEN(HL) to test the hypothesis that the participants with DRs (n = 4) would have elevated ACC thresholds relative to the normally hearing participants (n = 10) and participants with hearing impairment but without DRs (n = 12). They reported that the ACC thresholds were “around or above 12 dB SNR” for the DR group and statistically elevated relative to the threshold in normally hearing and hearing-impaired groups. Therefore, the authors suggested using the 12 dB SNR as a potential criterion for cochlear DR diagnosis, similar to the 10-dB criterion in the TEN(HL) test.

This approach presents several advantages: The testing is performed at the same level as the behavioural TEN(HL), so at a safe level, solving an issue noted in Markessis et al.^28^. It does not require prior information on electrophysiological or behavioural threshold to set up recording parameters. Since it requires 6 min to record an SNR condition, the test is fast to perform, making it tolerable for older participants. Finally, because it mimics the behavioural TEN(HL) test, it is easy to translate it for clinical applications. However, several issues need to be addressed first before this recommendation (i.e., 12 dB SNR rule) can be accepted for use in clinics with hearing-impaired patients with and without cochlear DRs. Firstly, Kang et al.^16^, did not specify a clear definition of the ACC threshold, which leaves significant room for error. Secondly, the criteria for accepting the presence of an ACC were not clearly defined. Although the ACC was categorised as present based to a visual decision and when the root mean square (RMS) amplitude of an ACC was 50% above the noise floor, the authors did not report the values of RMS or audiologists’ decision, thus it remains unclear how the single ACC was actually detected. It is good practice to state clearly a definition of the ACC presence and threshold (e.g.,^1^). In the behavioural TEN(HL) test, the thresholds are measured for each participant (e.g^.,26^). Therefore, the RMS for each waveform and participant is necessary. Thirdly, the ACC thresholds at 1 and 4 kHz for normally hearing and hearing-impaired participants were based on grand average values (^16^, cf. Figure 4 and Table 2). This poses a major problem as the focus should be on individual results. In particular, the clinician needs to know the TEN(HL) level and the ACC threshold for each participant at each test frequency and in each SNR to be able to form a meaningful diagnosis. Finally, the 12 dB SNR criterion seems to have been chosen arbitrarily as only four participants with cochlear DRs were tested with DRs starting at different frequencies and there is no evidence that the ACC threshold is frequency independent.

The aim of the current study was to address some of the issues identified in Kang et al.^16^. Specifically, we aimed to analyse all ACC waveforms at the single-participant level rather than at the grand average level, and to evaluate the presence of each single ACC using three analysis methods: i) RMS-based method as proposed by Kang et al.^16^, ii) SNR-based method as recommended by the British Society of Audiology (BSA^1^), iii) bootstrap-based method as described by Lv et al.^32^. We also aimed to assess the agreement between the methods and the repeatability of each of the methods used.

## Methods

### Participants

Participants were recruited via email through the Hearing Research Volunteer Database of the Manchester Centre for Audiology and Deafness and through the daily announcements of the Faculty of Biology, Medicine, and Health at the University of Manchester. A total of 23 participants aged 19–36 years took part in the experiments (11 males and 12 females). Participants were required to have clinically normal or near-normal hearing thresholds between 0.5 and 8 kHz, i.e., below 20 dB HL. The participants had no history of hearing problems or medical interventions.

The study was approved by the School of Health Sciences Ethics Committee, University of Manchester, and performed in accordance with the Declaration of Helsinki. All participants provided their written informed consent to participate in this study, and they were compensated for their time. All methods were carried out in accordance with relevant guidelines and regulations.

The sample size was limited by time and funding constraints. However, it was estimated to be sufficient to provide meaningful data, with further increases unlikely to yielded significant additional insights.

### Hearing evaluation

Otoscopy and the pure-tone air-conduction threshold audiometry were conducted prior to the experiments. The hearing threshold was performed in accordance with the BSA recommendation^33^. The participants were seated in a soundproof booth. The hearing threshold was measured using a GSI 61 audiometer (Grason-Stadler, Eden Prairie, MN, USA) through Etymotic Research (ER) 3 Insert Earphones. Because of the TEN(HL) test procedure (described in Sect. “ACC Processing”), the pure-tone audiometry was performed using 2-dB steps, the signal was decreased by 4 dB with correct response and the signal was increased by 2 dB with each incorrect response. For each tested frequency (between 0.5–8 kHz) the initial signal level was set to 40 dB HL.

### TEN(HL) test

Pure tones of 1 and 4 kHz were presented in TEN(HL)^26^ of 60 dB HL/Equivalent Rectangular Bandwidth (ERB_N_) — the same value as in Kang et al.^16^, which correspond to 81.2 and 86.6 dB HL at 1 and 4 kHz, respectively. Both the TEN(HL) and the signal were played from a CD using a Marantz CD5001 CD player, connected to the Grason-Stadler GSI 61 audiometer through ER3 Insert Earphones. The TEN level was fixed and the level of the signal was changing. Specifically, The signal level was manually changed using adaptive 2-down/1-up method, starting at 70 dB HL/ERB_N_. If the participant indicated the presence of the pure tone, the tone was decreased by 4 dB, otherwise the tone was increased by 2 dB.

### ACC recording

#### Stimulus

The stimuli were created by combining the TEN(HL) with a 1-kHz and a 4-kHz pure tone (TEN(HL)-Tone). The TEN(HL) lasted 1.5 s, the pure tone began at 1 s and lasted for 0.5 s. The pure tone had 5-ms raised-cosine rise/fall times. The inter-stimulus interval was 1.5 s. The stimuli were generated and presented using MATLAB (R2013a, 8.1.604, 64-bit). The TEN(HL)-Tone stimuli were generated at six different SNRs, ranging from 0 to 15 dB, with a 3-dB step size.

The TEN(HL)-Tone stimulus can evoke three responses^7^: i. onset response that is triggered by the onset of the TEN(HL); ii. ACC response that is triggered by the onset of Tone in the ongoing TEN(HL); iii. offset response that is triggered by the offset of the TEN(HL)-Tone stimulus. The duration of each stimulus component (TEN, pure tone and inter-stimulus interval) was carefully selected to avoid interference between these responses, thus allowing us to focus only on the ACC response.

#### ACC recording

The electrophysiological responses were recorded using a Biosemi EEG system with four active (preamplified) electrodes, AD-box with rechargeable battery and USB2 Receiver connected to the computer. The data were recorded with Biosemi software and on a personal computer (HP with dual processor Intel i7 6700 at 3.40 GHz, RAM of 32 GB and Windows 7). Low pass filtering was performed by the hardware with a fifth-order cascaded integrator-comb filter response, with a −3 dB point at 410 Hz (i.e., 2048/5). The electrodes were mounted on the participants’ head using conductive paste (TEN20) and surgical tape. The recording electrodes were placed at Cz, ipsilateral (i.e., testing ear) mastoid, and contralateral mastoid. The ground (i.e., CMS/DRL for the Biosemi) electrodes were on the forehead. Stable voltages of at least ± 40 mV, measured between CMS and each active electrode, were obtained. Eyeblinks were not monitored. The sampling rate of the EEG was 2048 Hz. The stimuli were presented through the inserts E-A-RTONE 3 A, which were electrically unshielded, via Focusrite Scarlett 2i2 soundcard routed connected to the Grason-Stadler GSI 61 audiometer.

#### ACC procedure

The ACC recording was performed in a double-wall soundproof booth. For each combination of frequency and SNR, the ACC was recorded twice. The participants were comfortably sitting in a reclining chair and were asked to ignore the stimuli and focus on watching silent, closed-captioned movie, or on reading a book or an article. The participants were monitored by the experimenter throughout the session to ensure they did not fall asleep or let their attention deteriorate significantly. Only one ear was tested in this experiment, and it was chosen based on the audiogram: the ear with either the best or less variable audiogram was selected, which was the right ear 12 times and the left ear 10 times. The contralateral (non-test) ear was plugged with a yellow foam earplug. The signals were played using constant stimuli method – each SNR combination, starting from 15 to 0 dB SNR with 3 dB steps, was presented to the participant with 120 repetitions (sweeps). Half of the participants started with 1-kHz pure tone in TEN(HL) while the other half started their test session with 4-kHz pure tone in TEN(HL). Each frequency measurement lasted about 36 min (i.e., 3 s × 120 sweeps × 6 SNRs); the whole session (including otoscopy and pure-tone audiometry) took no more than 3 h. The participants were offered a rest break after all the measures for a single frequency were collected.

#### ACC processing

**Fig. 1:**
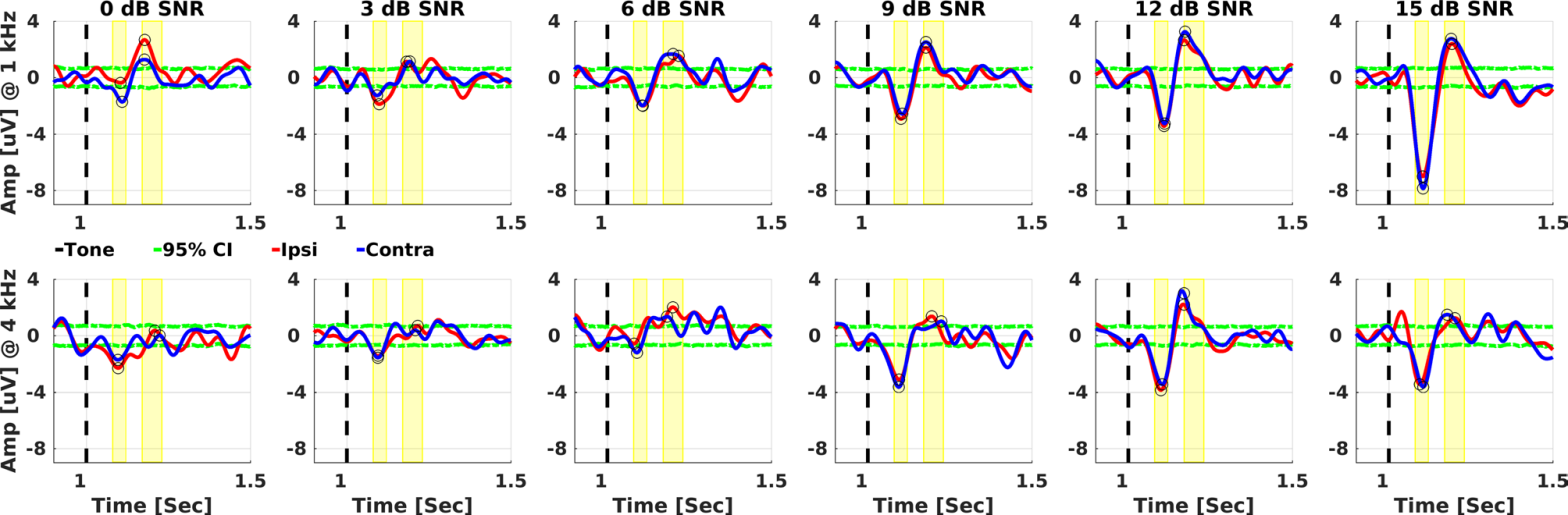
An example of electrophysiological Acoustic Change Complex (ACC) evoked by Threshold-Equalizing Noise [TEN(HL)] with a 1-kHz (first row) and a 4-kHz (second row) pure tone at 6 signal-to-noise ratios (SNRs). Yellow rectangles and black circles indicate the N1 and P2 peaks. Green dashed lines indicate the 95% confidence interval obtained via the bootstrap method. For graphical purposes, ACC responses were baseline corrected by subtracting the mean amplitude from −0.9 to 1 s.

Electrophysiological data were analysed offline in MATLAB for each frequency and SNR combination. The continuous EEG data of the two mastoids were referenced to Cz and then bandpass filtered from 1 to 30 Hz using a 3rd-order Chebyshev Type II filter with ripple of 30 dB. Considering the TEN(HL) onset occurred at 0 s, 120 epochs of 2.6 s, with 0.1 s pre-stimulus baseline were created. Epochs were baseline corrected by subtracting the mean amplitude calculated over the time period from −0.1 to 0 s. Epochs with their root-mean-square (RMS) values larger than two standard deviations of their mean value were rejected from further analysis^34^. The remaining epochs were averaged. The N1-P2 waves of the ACC were identified separately for the two mastoids (Fig. 1). The waves were obtained automatically but visually inspected by two authors. The N1 peaks were the most negative potential at 1.080–1.120 s (i.e., 80–120 ms post pure-tone onset), while the P2 peaks were the most positive potential at 1.170–1.230 s (i.e., 170–230 ms post pure-tone onset).

### ACC detection methods

Three different methods were used to categorise the ACC-responses as present or absent for each frequency and SNR. The inconclusive category was assigned to all waveforms that were rejected from further analysis as described in 2.5.4.

#### RMS method

The first method was the same as proposed by Kang et al.^16^. The RMS value was estimated for N1-P2 amplitude as well as for noise floor. N1-P2 amplitude was the peak-to-peak amplitude of ACC recorded between 80 and 230 ms after the pure-tone onset and the noise floor was calculated between 80 and 230 ms after the signal offset, i.e., during a no-stimulus period.

The ACC was accepted as present when the RMS of N1-P2 amplitude was at least 50% higher than the RMS of a noise floor (N1-P2 RMS ≥ 1.5 noise floor RMS).

#### BSA method

The second method was based on BSA recommendation^1^ for the Cortical Auditory Evoked Potentials (but see also^35^), two repetitions of ACC recording were compared (e.g. two recordings of ACC evoked by 1-kHz pure tone presented in TEN(HL) at 10 dB SNR). To estimate the “SNR” of the repeated ACC recording the N1-P2 amplitude was regarded as the “signal” value while the “noise” floor was the mean of the difference between the two recordings represented by the standard deviation of the two recordings. Since two repetitions of recordings obtained using the same SNR are compared (e.g., 1 kHz at 10 dB SNR), the relative SNR might be evaluated, which is the mean N1-P2 amplitude of both repetitions divided by standard deviation of these amplitudes.

According to this method, the ACC response was significant if the relative “SNR” was > 3 dB. This means that the ACC potential was present in the recorded EEG and thus the pure tone was detected in the TEN(HL).

#### Bootstrap method

This method is fully described in Lv et al.^32^. The bootstrap approach estimates the probability that visually-identified ACC response was present in the EEG. In this method, all 120 epochs for both ipsilateral and contralateral conditions were concatenated to form a single time series. From this series, 240 new epochs were randomly generated. This process was repeated 499 times to calculate the variance in the N1-P2 response to noise floor ratio from the resampled data. The confidence intervals of the estimate, i.e., the probability that the obtained signal represents the ACC waveform rather than random data variation, are then determined. In case of ACC measurements, the bootstrap method can confirm that the N1-P2 peaks are detected with 95% confidence. The ACC is categorised as present when the results fall outside the 95% confidence interval (see green dashed lines in the Fig. 1).

### ACC threshold estimation

#### Definitions

The ACC-threshold was defined as the lowest level of pure tone at which an ACC-response was present, with an ACC-response absent at a level of 3 dB below this level and present at 3 dB above it.

#### Statistical analysis

**Fig. 2:**
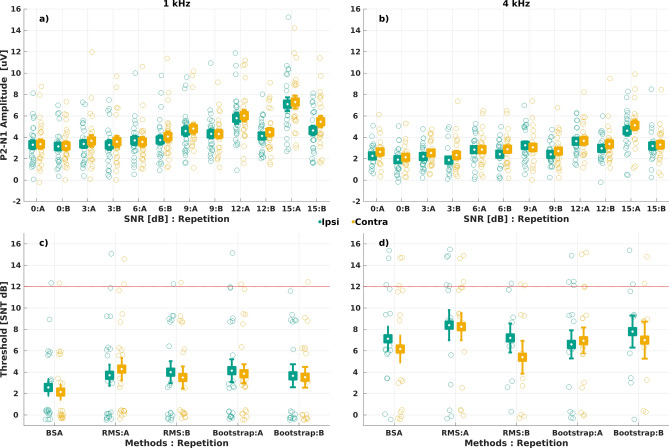
ACC amplitude (i.e., P2-N1) as a function of SNR at 1 kHz (**a**) and at 4 kHz (**b**). ACC-thresholds for the three different methods at 1 kHz (**c**) and at 4 kHz (**d**). Error bars show the mean and standard deviation. Open circles represent individual data points. Red dotted line at 12 dB SNR is the threshold proposed by Kang et al.^16^ as a cut-off criterion to diagnose cochlear DRs using ACC.

Figure 2 shows the individual ACC-thresholds determined using three ACC-response detection methods: 1) RMS method, 2) BSA method, and 3) Bootstrap method.

The three ACC-threshold estimation methods were compared using Bland–Altman plots^36,37^ and the concordance correlation coefficient (CCC^38,39^). Bland & Altman proposed the limits of agreement (LoA) as a statistical method to compare two clinical measurement methods and to assess the repeatability of one clinical measurement method^36,37^. A Bland–Altman plot is created by calculating the mean and standard deviation (SD) of the difference of two measurements for each participant, estimating the 95% LoA from the mean of the two measurements and adding (upper limit) or subtracting (lower limit) 1.96 × SD. It is expected that the 95% LoA include 95% of differences between the two measurement methods. In the case of repeated measurements of one clinical method, assuming that the true value (i.e. ACC thresholds) is constant, a correction for the variance is applied^37^.

The reliability of the ACC thresholds estimated using the three methods was assessed using the reproducibility index CCC, which evaluates the agreement between two methods by measuring the variation from the equality line, so the index combines measures of both precision and accuracy, ranging from −1 to 1^38^. CCC was calculated in R using the *epiR* package^40^.

The agreements between the methods were calculated using only the first repetition for the RMS and Bootstrap methods, and the overall ACC-thresholds for the BSA method. The rationale of using only the first repetition was that to decide whether a method is clinically valid (i.e., agree sufficiently with the established method), only one measurement of the ACC-threshold would be required. In the case of the RMS and Bootstrap methods, the test–retest or repeatability between the repetitions was estimated via Bland–Altman plot. The coefficient of repeatability was calculated as twice the SD of differences between the repetitions^36^.

For the agreement and the repeatability analyses, the ipsi- and contra-lateral ACC thresholds were considered as repeated measurements. This assumption is considered valid because the contralateral ACC thresholds of a method were compared against the contralateral ACC thresholds of another method.

## Results

### TEN test

The TEN thresholds expressed as the Signal-to-TEN Ratio (STR^41^) ranged from −6 to 2 dB (green circles in Fig. 5), confirming that no participants exceeded the 10 dB rule, i.e., no participants had cochlear DRs. The comparison of TEN and ACC thresholds is provided in the Supplement.

### ACC amplitudes as a function of SNR

The ACC-response amplitudes as a function of SNR for 1 and 4 kHz, and ACC thresholds at 1 and 4 kHz are shown in Fig. 2. Due to attrition, human error, or equipment faults, the number of ACC recordings was 513 instead of 552 (i.e., 23 participants × 2 frequencies × 6 SNRs × 2 repetitions). Among these, 85 recordings had a high level of noise and artefact resulting in ACC response not present. The number of thresholds was 332 instead of 460 (i.e., 23 participants × 2 frequencies × 5 methods/repetitions x ipsi- and contra-lateral ear), due to a lack of ACC response and, in general, the inability to determine a threshold in the contralateral ear. The mean (and range) ACC-thresholds estimated using the RMS method were 3.88 (0–15) dB SNR at 1 kHz and 7.53 (0–15) dB SNR at 4 kHz. Using the BSA method, the mean (and range) ACC-thresholds were 2.36 (0–12) dB SNR at 1 kHz and 6.62 (0–15) dB SNR at 4 kHz. For the Bootstrap method, the mean (and range) ACC-thresholds were 3.82 (0–15) dB SNR at 1 kHz and 7.02 (0–15) dB SNR at 4 kHz.

### Agreement between the three methods of ACC-threshold estimation

**Fig. 3:**
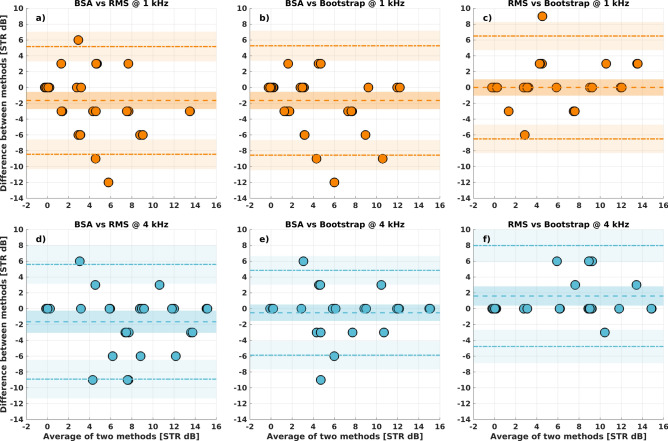
Agreement between the three methods presented by difference between ACC threshold values obtained for each pair of methods as a function of average of these methods at 1 (first row, in orange) and 4 kHz (second row, in blue). Dashed lines are the bias; dash-dot lines are the upper and lower the limits of agreements (LoAs); shadowed areas around the lines are the confidence intervals.

Figure 3 shows the Bland–Altman plots of the difference between the mean ACC-thresholds estimated using the following methods: BSA vs RMS, BSA vs Bootstrap, and RMS vs Bootstrap. The bias and the limits of agreement (LoA) with 95% CI are shown in Table 1.

**Table 1:** Comparison of each method in terms of bias and limits of agreement (LoA) at 1 and 4 kHz. Lower CI and Upper CI represent lower and upper 95% confidence intervals for each value.

Comp	Val	Lower CI	Upper CI
BSA vs RMS @ 1 kHz			
bias	−1.64	−2.72	−0.56
lower LoA	−8.45	−10.32	−6.57
upper LoA	5.16	3.29	7.04
BSA vs Bootstrap @ 1 kHz			
bias	−1.64	−2.74	−0.54
lower LoA	−8.56	−10.46	−6.65
upper LoA	5.27	3.37	7.17
RMS vs Bootstrap @ 1 kHz			
bias	0	−1.03	1.03
lower LoA	−6.50	−8.29	−4.71
upper LoA	6.50	4.71	8.29
BSA vs RNS @ 4 kHz			
bias	−1.66	−3.06	−0.25
lower LoA	−8.90	−11.34	−6.47
upper LoA	5.59	3.16	8.03
BSA vs Bootstrap @ 4 kHz			
bias	−0.52	−1.56	0.52
lower LoA	−5.88	−7.68	−4.08
upper LoA	4.84	3.04	6.65
RMS vs Bootstrap @ 4 kHz			
bias	1.60	0.38	2.82
lower LoA	−4.78	−6.88	−2.67
upper LoA	7.98	5.87	10.08

The CCC values for BSA vs RMS at 1 and 4 kHz were 0.59 (CI = 0.39/0.74) and 0.70 (CI = 0.47/0.84), respectively; for BSA vs Bootstrap at 1 and 4 kHz, the values were 0.62 (CI = 0.42/0.76) and 0.84 (CI = 0.68/0.92), respectively; for RMS vs Bootstrap at 1 and 4 kHz, they were 0.73 (CI = 0.55/0.85) and 0.76 (CI = 0.57/0.87), respectively.

### Repeatability of ACC-thresholds estimated using RMS and bootstrap methods

**Fig. 4:**
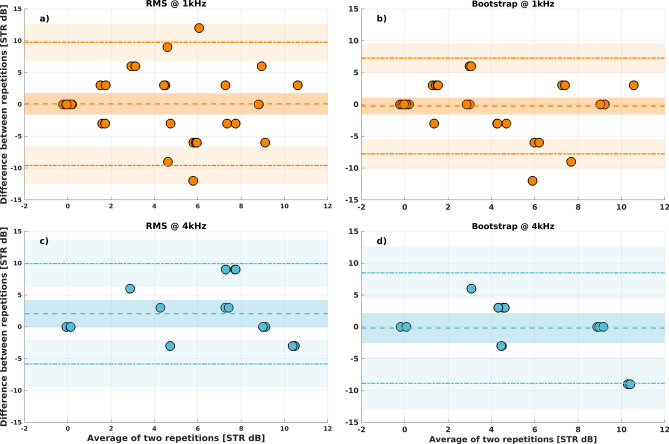
Repeatability of 1- (first row, in orange) and 4-kHz (second row, in blue) ACC-thresholds obtained using RMS and Bootstrap methods. Dashed lines depict the bias; dash-dot lines are the upper and lower LoAs; shadow areas show confidence intervals.

Figure 4 shows the Bland–Altman plots for assessing the repeatability of ACC-threshold estimation using the RMS and Bootstrap methods over the two repetitions.

The bias and the limits of agreement (LoA) with 95% CI are shown in Table 2. For the RMS method, the coefficients of repeatability were 9.87 and 8.05 dB SNR at 1 and 4 kHz, respectively. For the Bootstrap method, the coefficients of repeatability were 7.67 and 8.86 dB SNR at 1 and 4 kHz, respectively.

**Table 2:** Repeatability of ACC-threshold estimation using RMS and bootstrap methods, in terms of bias and limits of agreement (LoA), at 1 and 4 kHz. Lower CI and Upper CI represent lower and upper confidence intervals for each value.

Comp	Val	Lower CI	Upper Ci
RMS @ 1 kHz			
bias	0.08	−1.59	1.75
lower LoA	−9.59	−12.48	−6.70
upper LoA	9.76	6.87	12.65
Bootstrap @ 1 kHz			
bias	−0.26	−1.57	1.06
lower LoA	−7.77	−10.05	−5.49
upper LoA	7.26	4.98	9.54
RMS @ 4 kHz			
bias	2.06	−0.08	4.21
lower LoA	−5.82	−9.54	−2.11
upper LoA	9.95	6.23	13.66
Bootstrap @ 4 kHz			
bias	−0.19	−2.55	2.17
lower LoA	−8.87	−12.96	−4.78
upper LoA	8.50	4.41	12.58

## Discussion

### Comparison of ACC detection methods

The aim of the first part of the experiment was to establish the most reliable method to determine the ACC threshold in normal-hearing participants. Extending earlier work^16^, the present study used three different methods to detect ACC responses and provide analysis to support our choice. Although the BSA method is regarded as the “gold standard” in audiology testing, two other methods were introduced and compared against it to ensure that the most efficient method was chosen for further ACC examination. The results used for the analysis were presented for each participant separately, which allowed for more detailed comparison of the methods.

The Bland–Altman plots presented in Fig. 3 and the CCC values demonstrated better agreement between BSA and Bootstrap method than between BSA and RMS method. For comparison between BSA and Bootstrap method, the 95% confidence intervals and LoA provided smaller dispersion from the mean value than when the BSA was compared to RMS method. This was true in particular at 4 kHz, the difference between the RMS and Bootstrap methods compared to the BSA method was less apparent at 1 kHz.

For each plot in Fig. 2, the data were less scattered at 4 kHz than at 1 kHz. In first approximation, the methods gave similar results at 4 kHz, whereas the differences were more pronounced at 1 kHz. It must be also indicated that the greatest agreement was obtained for RMS vs Bootstrap method. The reason for this, however, could have been the number of repetitions carried out for each method, which is discussed further in the next paragraph.

The Bland–Altman plots were also used for assessing the repeatability of the RMS and Bootstrap methods over the two repetitions (Fig. 4). The RMS method provided worse repeatability than the Bootstrap method and thus it was concluded not to be the most reliable choice for assessing the ACC threshold. Since the RMS and Bootstrap methods were the new/proposed approaches, we used the two repetitions to evaluate their repeatability. The evaluation of repeatability for the BSA method was not performed because it would have required four repetitions of ACC recordings at each SNR and each frequency, which, due to time constraints, we did not conduct.

In terms of similarity, the Bootstrap method provided comparable results to the BSA method. At the same time, the repeatability of results for two repetitions of Bootstrap method was better than those of the RMS method. For this reason, we concluded that BSA and Bootstrap methods gave the optimal ACC-threshold estimations. Moreover, Bootstrap method is twice as fast to administer than the BSA method, which is a huge advantage when testing infants, children and adults who cannot provide behavioural responses.

### ACC amplitude and thresholds change with SNR and frequency

According to He et al.^10^, the latency of the ACC signal is not as effective parameter in threshold evaluation as N1-P2 amplitude of the waveform. Therefore, only the amplitude of the ACC evoked by a combination of SNRs and frequencies was investigated in this study. The value of N1-P2 increased with an increase in the SNR value (Fig. 2, panels a and b). This pattern was observed in both the first and second repetitions of the test at the same SNRs. Similarly, Billings et al.^13^ found that ACC amplitude varied across SNRs (ranging from −20 to + 10 dB). They also showed that the presence of noise was essential for detecting this change, as no amplitude differences were observed when signal levels varied in quiet conditions. As the ACC response in our experiment was elicited by a change in signal timbre, namely, change not only in amplitude, but in frequency as well (a pure tone of different signal level added to a steady noise), it is in agreement with previous findings that a change in signal frequency but not intensity itself is responsible for change in ACC amplitude^9^. However, it should be pointed out that the values of N1-P2 amplitude for the second repetition of ACC measurement were generally lower. It might have been due to neural adaptation/habituation effects as both presentations of the same SNR were made during one session^42^.

The direction of the presentation pattern was always downward, meaning the first SNR presented was 15 dB SNR and the value was decreasing down to 0 dB SNR. Although our results were consistent with findings of He et al.^10^, future research should evaluate whether, and to what extent, the N1-P2 amplitude depends on the presentation order.

The same trend was observed for both frequencies (1 and 4 kHz) but the value of the N1-P2 amplitude for all SNRs was lower for the 4-kHz pure tone. This led to higher ACC thresholds at 4 kHz across all methods (Fig. 2, panels c and d). The outcome of these findings is that the frequency of the signal must be taken into consideration when it comes to threshold evaluation because otherwise some false positive as well as false negatives results might occur in the data. This is also in agreement with the findings of Dimitrijevic et al.^9^, who compared the auditory cortical potentials as a function of spectral changes for pure tones at 0.25 and 4 kHz. They showed that the cortical response changes with the frequency of signal presented, and that the amplitude of the potential is higher for the lower frequency.

### TEN vs. ACC thresholds

**Fig. 5:**
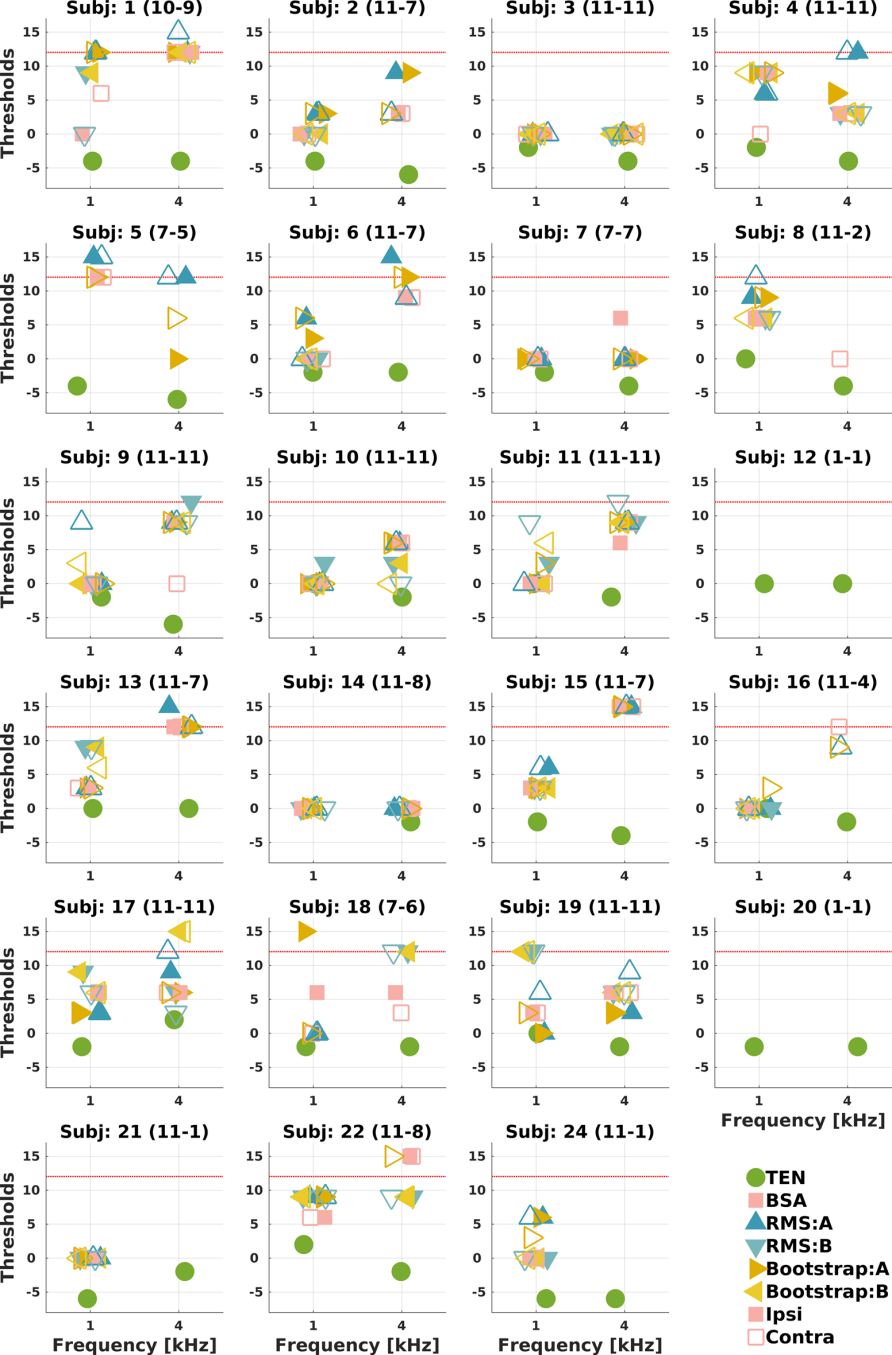
ACC-thresholds expressed as Signal-to-TEN(HL) ratio in dB at threshold for each of the 23 participants. Green circles: behavioral TEN(HL) thresholds; pink squares: ACC-thresholds determined using the BSA Method^1;^ dark-blue upward-pointing triangles and light-blue downward-pointing triangles: ACC-thresholds determined using the RMS method^16^ for the first (i.e. A) and second (i.e. B) repetition, respectively; dark-yellow right-pointing triangles and light-yellow left-pointing triangles: ACC-thresholds determined using the Bootstrap method^32^ for the first (i.e. A) and second (i.e. B) repetition, respectively. Full and open symbols denote ipsi- and contra-lateral ACC, respectively. Number of thresholds are in the brackets. Red dotted line at 12 dB SNR is the threshold proposed by Kang et al.^16^ as a cut-off criterion to diagnose cochlear DRs using ACC. For the clarity of the figure some horizontal/vertical jitter was used.

In order to use the objective ACC test in clinics, the ACC thresholds for both TEN and ACC test must show good agreement. The results of this study are the perfect example of how important it is to establish the correct ACC threshold. Figure 5 shows the TEN thresholds and the ACC thresholds obtained using the three methods for each participant. Since the ACC thresholds were typically higher than the TEN thresholds, ‘correction’ factors should be established to accurately predict TEN thresholds from ACC thresholds. The use of ‘correction’ factors would be similar to those applied to auditory brainstem responses to estimate hearing thresholds^43^. Kang et al.^16^ proposed 12 dB SNR rule to diagnose DRs using ACC, that is, ACC threshold at or above 12 dB SNR would indicate a presence of a DR. In the current study, no DRs were diagnosed using behavioural TEN(HL), as it is shown in Fig. 2. Although the mean value of the ACC-thresholds (for all presented methods) in our experiment fell far below the 12 dB SNR, when analysing individual participants’ ACC-thresholds, some were higher than the 12 dB SNR (Fig. 5: Subj 1, 4, 5, 6, 9, 13, 15, 17, 18, 19, 22). Since all our participants had normal hearing and thus no cochlear DRs, clearly the proposed 12 dB SNR rule would incorrectly diagnose these individuals as having cochlear DRs. Thus, the 12 dB SNR ACC-threshold rule for diagnosing DRs should be reviewed. Furthermore, maturational effects on the ACC^44^ should be accounted for when establishing rules for diagnosing DRs in paediatric population. A related issue is that arousal states vary more in infants and children than in adults.

Some inter-subject variability was observed for the ACC threshold. In general, ACC waveforms vary substantially as a function of participant’s state of arousal^1,2^. In the current study, the state of arousal was monitored and maintained, but drowsiness might have had an effect on the ACC waveforms and, in turn, on the ACC thresholds. This may be particularly true for the second repetition as the ACC amplitude was lower than the first recording. The second repetition is required only the BSA method, and it is not needed for the Bootstrap method. So, using the Bootstrap method, participants’ drowsiness may be avoided. However, the interaction between ACC amplitude and stimulus intensity is characterised by intra-and inter-individual variability^2^. Furthermore, Billings et al.^45^ reported strong variability in hearing-impaired adults.

Only normal-hearing participants were included in the current study. Mathew et al.^15^ observed that ACCs evoked by a frequency shifted complex tone are somewhat more robust in normal-hearing participants than those with sensorineural hearing loss.

Since ACC amplitude is weaker and more variable in hearing-impaired participants, it is important that future work should evaluate ACC thresholds in hearing-impaired participants, both with and without DRs, and the sensitivity and specificity of the Bootstrap method is assessed.

## Conclusions

The development of the objective test to measure cochlear DRs must take into consideration many factors that include repeatability of the test results, frequency-dependence, agreement between electrophysiological and behavioural test results, method of results evaluation and signal presentation pattern.

Three different methods of the ACC threshold evaluation were assessed, and the Bootstrap method was found to be most effective and reliable way to measure ACC thresholds.

The results presented for 23 participants indicated that there is a significant relationship between the ACC threshold, amplitude and signal frequency.

It is not yet clear to what extent the results of the ACC threshold are dependent on signal presentation order or neural adaptation/habituation effects.

To establish the objective test to detect DRs, many different frequencies as well as different signal presentation patterns should be further examined. It must be, however, emphasised that the ACC test development requires a large group of patients with DRs to be tested before this method can be recommended for use in clinics.

## Supplementary Information

Supplementary Information.

## Data Availability

The datasets used and/or analysed during the current study available from the corresponding author on reasonable request. All data generated or analysed during this study are included in this published article.
