# Supplementary material for: The acoustic change complex as a diagnostic tool for cochlear dead regions evaluated in normally hearing adults
Source: Sci Rep. 2025 Jul 7;15:24160. doi: 10.1038/s41598-025-02093-w (PMC12230141; doi:10.1038/s41598-025-02093-w)
Supplement: Supplementary file 1 — Supplementary Information. [file 41598_2025_2093_MOESM1_ESM.pdf]

The Acoustic Change Complex as a Diagnostic Tool for Cochlear Dead Regions: A Study in Normally Hearing Adults

Supplement

Anna Schelenz, Emanuele Perugia, Lin Wu, Ewa Skrodzka, and Karolina Kluk

List of Figures

S1 Correlations 2
S2 Agreement 3

List of Tables

S1 Agreement 4

Correlations

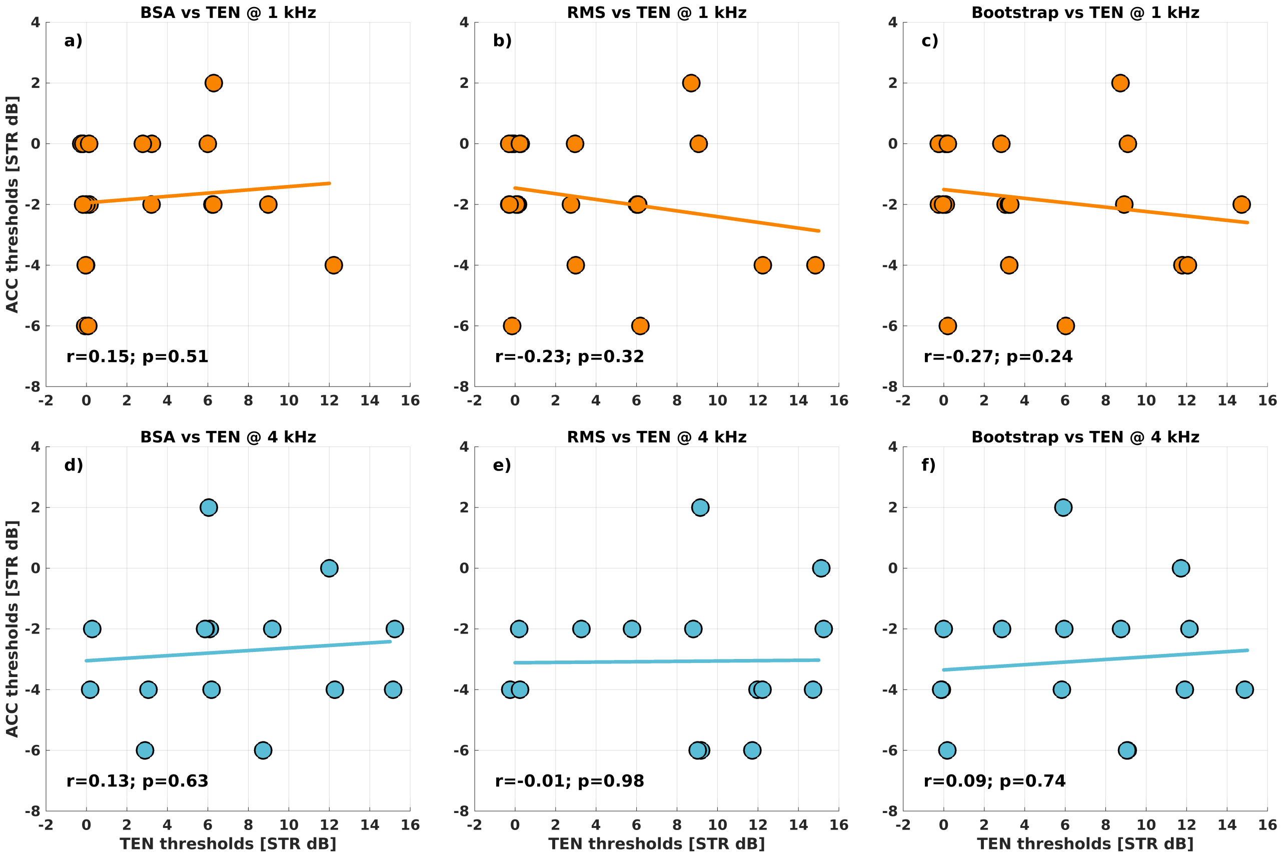

Figure S1: Scatter plots with Spearman's rho between TEN and ipsilateral ACC thresholds obtained using BSA, RMS, and Bootstrap methods.

Agreement

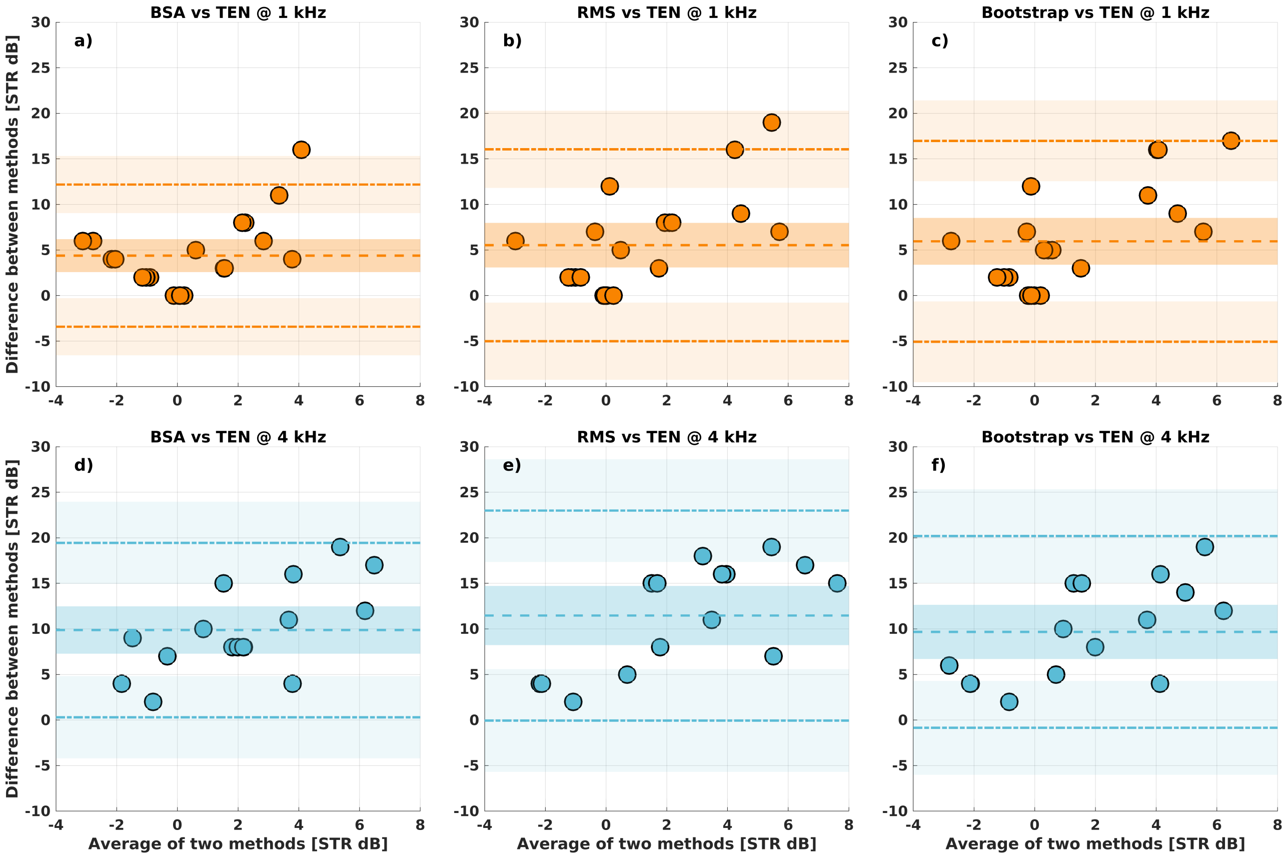

Figure S2: Agreement between TEN and ipsilateral ACC thresholds obtained using BSA, RMS, and Bootstrap methods.

Table S1: Agreement between TEN and ipsilateral ACC thresholds obtained using BSA, RMS, and Bootstrap methods in terms of bias and limits of agreement (LoA), at 1 and 4 kHz. Lower CI and Upper CI represent lower and upper confidence intervals for each value.

Comp	Val	LowerCI	UpperCI
BSA vs TEN @ 1 kHz			
bias	4.38	2.57	6.19
lower LoA	-3.42	-6.56	-0.28
upper LoA	12.18	9.04	15.32
RMS vs TEN @ 1 kHz			
bias	5.52	3.08	7.97
lower LoA	-5.01	-9.24	-0.77
upper LoA	16.05	11.82	20.29
Bootstrap vs TEN @ 1 kHz			
bias	5.95	3.39	8.51
lower LoA	-5.07	-9.51	-0.64
upper LoA	16.98	12.54	21.41
BSA vs TEN @ 4 kHz			
bias	9.88	7.27	12.48
lower LoA	0.30	-4.20	4.81
upper LoA	19.45	14.94	23.95
RMS vs TEN @ 4 kHz			
bias	11.47	8.21	14.72
lower LoA	-0.05	-5.69	5.58
upper LoA	22.99	17.35	28.63
Bootstrap vs TEN @ 4 kHz			
bias	9.67	6.69	12.64
lower LoA	-0.85	-6.00	4.29
upper LoA	20.19	15.04	25.34
